# V-ATPase Is Involved in Silkworm Defense Response against *Bombyx mori* Nucleopolyhedrovirus

**DOI:** 10.1371/journal.pone.0064962

**Published:** 2013-06-18

**Authors:** Peng Lü, Hengchuan Xia, Lu Gao, Ye Pan, Yong Wang, Xin Cheng, Honggang Lü, Feng Lin, Liang Chen, Qin Yao, Xiaoyong Liu, Qi Tang, Keping Chen

**Affiliations:** 1 School of Food and Biological Engineering, Jiangsu University, Zhenjiang, Jiangsu, PR China; 2 Institute of Life Sciences, Jiangsu University, Zhenjiang, Jiangsu, PR China; 3 School of Medical Science and Laboratory Medicine, Jiangsu University, Zhenjiang, Jiangsu, PR China; 4 The Laboratory Animal Research Center, Jiangsu University, Zhenjiang, Jiangsu, PR China; 5 Nanchang Key Laboratory of Applied Fermentation Technology, Jiangxi Agricultural University, Nanchang, PR China; 6 Zhejiang Institute of Freshwater Fisheries, Huzhou, Zhejiang, PR China; Swedish University of Agricultural Sciences, Sweden

## Abstract

Silkworms are usually susceptible to the infection of *Bombyx mori (B. mori) nucleopolyhedrovirus* (BmNPV), which can cause significant economic loss. However, some silkworm strains are identified to be highly resistant to BmNPV. To explore the silkworm genes involved in this resistance in the present study, we performed comparative real-time PCR, ATPase assay, over-expression and sub-cellular localization experiments. We found that when inoculated with BmNPV both the expression and activity of V-ATPase were significantly up-regulated in the midgut column cells (not the goblet cells) of BmNPV-resistant strains (NB and BC8), the main sites for the first step of BmNPV invasion, but not in those of a BmNPV-susceptible strain 306. Furthermore, this up-regulation mainly took place during the first 24 hours post inoculation (hpi), the essential period required for establishment of virus infection, and then was down-regulated to normal levels. Amazingly, transient over-expression of V-ATPase c subunit in BmNPV-infected silkworm cells could significantly inhibit BmNPV proliferation. To our knowledge this is the first report demonstrating clearly that V-ATPase is indeed involved in the defense response against BmNPV. Our data further suggests that prompt and potent regulation of V-ATPase may be essential for execution of this response, which may enable fast acidification of endosomes and/or lysosomes to render them competent for degradation of invading viruses.

## Introduction

The silkworm (*B. mori*) has long been raised as a beneficial insect in sericulture industry and a model insect of Lepidoptera in research. The sericulture often suffers huge economic loss caused by *B. mori* nucleopolyhedrovirus (BmNPV). Most silkworms are susceptible to BmNPV infection, which can cause devastating consequence due to lack of highly specific and effective pesticides. However, some strains have been isolated and characterized to be resistant to BmNPV [1], [2]. Certain silkworm metabolites and proteins have also been shown to possess antiviral activity against infection of BmNPV [3], [4], [5], [6], but the mechanism of *B. mori* defense response against BmNPV remains largely unknown.

It is well known that endosome system participates in the cellular entry of BmNPV. For example, the Baculovirus budded viruses (BVs) enter cell via clathrin-mediated endocytosis [7]. Once inside the endosome, the virus encoded gp64 protein can be enabled by the acidic environment to promote the membrane fusion between the virus and endosome to release the virons into the cytoplasm [8], [9], [10]. Obviously, the normal function of vacuolar-type H^+^-ATPase (V-ATPase) is required in this process to provide the acidic environment. V-ATPases locate in various endomembrane systems and plasma membranes [11], [12]. They are multi-subunit complexes organized into a peripheral domain (V_1_) responsible for ATP hydrolysis and an integral domain (V_0_) that transports protons across membranes [13], [14], [15], and are essential for pH regulation of the intracellular compartments, the cytoplasm and the extracellular space [16], [17], [18]. To our surprise, our preliminary proteomic analysis showed the expression of V-ATPase was up-regulated in BmNPV-resistant strain NB (unpublished data), indicating that V-ATPase may also play a role in the silkworm defense response against BmNPV.

In this study, we chose the c and B subunits to study the interaction between V-ATPase and anti-BmNPV, because both of them have already been sub-cloned [19], [20], and c subunit is the component for V_0_ domain and B for V_1_ domain. We performed comparative real-time PCR, ATPase assay, transient over-expression and sub-cellular localization experiments to further investigate the mechanism and regulation of V-ATPase in the silkworm anti-BmNPV response. Our data reveals for the first time that V-ATPase is indeed involved in the silkworm defense response against BmNPV. Our result also suggests that prompt and potent up-regulation of V-ATPase may be critical in this response, which may enable the fast acidification of endosomes and/or lysosomes to prepare them for efficient degradation of BmNPV viruses.

## Materials and Methods

### 1. Insect, cell line and virus

Silkworm ovary cell line BmN, BmNPV T3 isolate, BmNPV-susceptible silkworm strain 306 and -resistant strain NB are from our lab collection. The near-isogenic strain BC8, which is resistant to BmNPV infection but with similar genetic background to 306, was obtained as described by Yao et al. [21]. BmN cell line was cultured at 27°C in TC-100 medium supplemented with 10% fetal bovine serum (Gibco-BRL, Carlsbad) as described previously [22]. BmNPV was propagated in silkworm strain 306, and the occlusion bodies (OBs) of BmNPV were isolated and purified from the infected *B. mori* larvae as described by Summers and Smith [23]. Hemolymph-derived BVs were purified according to the method of Chen et al [24]. The numbers of obtained OBs and cells were examined using a hemocytometer under light microscope, and the titers of BVs were determined by a tissue culture infectious dose 50 (TCID50) method based on endpoint dilution [25].

### 2. Insect rearing and midgut collection

The silkworm larvae (306, NB and BC8) were reared on fresh mulberry at 27°C. Each newly molted 5th-instar larva was inoculated 1×10^6^ OBs per os (5 µl, enough to get 100% infection in the susceptible 306 strain) using an pipette, while the larvae were inoculated with same volume of phosphate buffer solution (PBS) were used as control. Then, at 0, 24, 48 and 72 hours post inoculation (hpi) they were dissected and the midguts were collected.

### 3. cDNA synthesis and Real-time PCR analysis

Total RNA was isolated using Trizol reagent (Invitrogen). The first-strand cDNA was synthesized with oligo (dT) primers and M-MLV reverse transcriptase (Promega) according to the manufacture's instructions. As shown in Table 1, the primers QVc-F and QVc-R were used to amplify the ORF of V-ATPase c subunit (Vc), and the QVB-F and QVB-R for V-ATPase B subunit (VB). The amplification of translation initiation factor 3 subunit 4 (TIF-3) was used as an internal control. PCR was carried out in triplicates using the following parameters: 2 min at 95°C, followed by 40 cycles of 30 s at 95°C, 30 s at 58°C and 20 s at 72°C.The quantity of PCR product is normalized using the threshold cycle (*C*_t_) value determined by the *TIF-3* amplification test and was analyzed by 2^−*ΔΔC*t^ method [26]. In each assay, the expression level is shown relative to the lowest expression level, which is arbitrarily set to one. Real-time PCR was carried out on Mx 3000P (Stratagene, San Diego, CA) system using the SYBR *Premix Ex Taq* Kit (Takara) according to the manufacturer's protocol in triplicate, and 0.2 µM of each primer in 25 µl final volume with the following amplification conditions: 2 min at 95°C for initial denaturation, followed by 40 cycles of 30 s at 95°C, 30 s at 58°C and 20 s at 72°C.

**Table 1:** Primers used in real-time PCR and construction of transient expression vectors.

Target gene	Accession no.	Primers (forward, reverse)^a^
*B. mori* V-ATPase c subunit	**EU082222**	QVc-F:5′CGGCGTCTGCTATCATCTTCA3′
		QVc-R:5′ CACGCACGCCTGCATCTC3′
		EVc-F:5′ggatccATGGCTGAAAATAATC3′
		EVc-R:5′ctcgagTTTTGTGTACAGGTAGA3′
*B. mori* V-ATPase B subunit	**EF107513**	QVB-F:5′TCACCCATCCCATTCCC3′
		QVB-R:5′CCTTGCCGATAGCGTAGC3′
		EVB-F:5′aggcctGAAAACTGAAAAATGGCAAAGGT3′
		EVB-R:5′gcggccgcATGGCGGGAGTCTCTCGG3′
*B. mori* translation initiation	**DQ443289**	TIF3-F:5′AGATGACGGGGAGCTTGATGGT3′
factor 3 subunit 4		TIF3-R:5′GAGGGCGGAATGTACTTGTTGC3′

a The restriction sites are underlined.

### 4. Preparation of crude midgut extracts and isolation of midgut plasma membranes

Midguts from *B. mori* were homogenized in 200 µl lysis buffer (16 mM Tris-HCl, 0.32 mM EGTA, 9.6 mM 2-mercaptoethanol, 0.1%C_12_E_10_, pH 8.1). After ultrasonication for 1 min, the crude midgut extract was stored at −20°C for ATPase assay. Preparation of midgut plasma membranes was performed as described by Wieczorek [27]. Briefly, the crude midgut extract was layered onto a discontinuous sucrose gradient, and after the centrifugation the target membrane fractions were diluted with buffer (5 mM Tris-HCl, 5 mM EGTA, pH 8.1), and then centrifuged at 100,000×g for 30 min at 4°C. The pellet was recovered and dissolved in lysis buffer as the plasma membrane fraction for ATPase assay. Protein concentration was determined with a BioRad *DC* Protein Assay kit (BioRad, USA).

### 5. ATPase assays

V-ATPase activity was determined as described by Huss et al. [28] with some modifications, and the concentrations of ATPase inhibitor bafilomycin A1 is 0.025 mM [29]. The final volume of 160 µl reaction buffer (4 µg protein, 50 mM Tris-Mops, 3 mM 2-mercaptoethanol, 1 mM MgCl2, 0.5 mM sodium azide, 20 mM KCl, 0.003% C12E10, 20 mM NaCl and 3 mM Tris-HCl, pH 8.1) was preincubated for 30 min with or without inhibitors at 27°C, then 1 mM Tris-ATP was added, and after an additional 10 min the reaction was stopped by placing the reaction tubes in liquid nitrogen. Aliquots of 30 µl of the resulting supernatant were transferred to the wells of a 96-well microtitre plate. The produced inorganic phosphate was measured at absorbance 710 nm after adding 100 µl 2% sodium citrate, 2% sodium arsenite, 2% acetic acid as described by Elandalloussi et al. [30],

### 6. Construction of transient expression vectors of V-ATPase c and B subunits

The *Bac-to-Bac* Baculovirus Expression System was used to transiently over-express the Vc and VB in BmN cells. To construct the GFP tagged plasmids, the *polh* promoter of pFastBac HTb (Gibco) was replaced by *ie-1* promoter, and the *egfp* was inserted into the *Xho* I/*Hind* III site to generate GFP vector pFastBacHTb-IE1p-EGFP. The *ie-1* promoter was amplified from BmNPV T3 genomic DNA using primers IE1-F and IE1-R, and *egfp* was amplified with primers EGFP-F and EGFP-R from plasmid of pEGFP-N1, which is donored by Prof. Chuanxi Zhang from Zhejiang University. The ORF of Vc subunit was amplified from silkworm midgut with primers EVc-F and EVc-R, and was sub-cloned to the *BamH* I/*Xho* I site of GFP vector without stop codon to construct pFastBacHTb-IE1p-Vc-EGFP. Similarly, the ORF of VB subunit was amplified with primers EVB-F and EVB-R, and was inserted into GFP vector at *Stu* I/*Not* I site without stop codon to obtain pFastBacHTb-IE1p-VB-EGFP. The primer sequences are listed in Table 1, and all constructs are verified by DNA sequencing.

### 7. The effects of V-ATPase c and B subunits on proliferation of BmNPV

BmN cells were infected with BVs at multiplicity of infection (MOI) of 2, and TC100 medium was used as mock control. 1 h after inoculation, the BmN cells were washed with fresh medium, and the BmNPV infected cells were transfected with plasmid pFastBacHTb-IE1p-Vc-EGFP or pFastBacHTb-IE1p-VB-EGFP by Lipofectin-mediated transfection method (Invitrogen). pFastBacHTb-IE1p-EGFP, the vector for above plasmids, was used for mock transfection. The BmNPV-infected cells alone were also used as control. Cells were examined with a fluorescence microscope (Leica, Germany) at 48 hours post transfection and the cells with different localization of Vc or VB were counted. Cell transfects were collected to check the expression of fusion protein by western blot as described by Zhao et al. [31] and V-ATPase activity as described above. The monoclonal antibody against EGFP and the FITC-conjugated secondary antibody were purchased from Proteintech Group Inc. The result was observed using FMBIO II (Hitachi, Japan). The rest was collected and centrifuged at 10,000×g for 5 min to remove cell debris prior to analysis of virus titers as described above.

### 8. Statistical analysis

The data presented is an average of three replicates with 6 larval midguts per replicate. A two-way analysis of variance (ANOVA) was used to compare the 306, NB and BC8 data as well as the PBS or BmNPV infected larval midguts. The pairwise comparisons for various parameters (relative expression levels and V-ATPase activities) among silkworm lines (306, NB and BC8) was done after post-hoc analysis using Tukey's multiple comparison test of the correspongding treatment factor using main effects in a one-way ANOVA. The significance between PBS and BmNPV inoculated groups was estimated by student's t-test.

## Results

### 1. Expression profile of V-ATPase c and B subunits in silkworms inoculated with BmNPV

**Figure 1:**
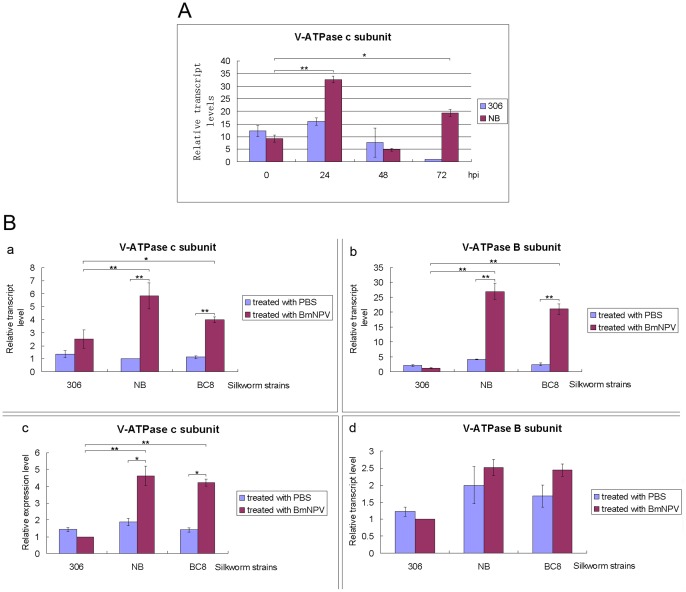
Real-time PCR analysis of expression profiles of V-ATPase c and B subunits in midguts of silkworm strain 306, NB and BC8 inoculated with BmNPV. The values for relative transcript levels of V-ATPase c and B subunit were calculated as described in Materials and Methods, and triple experiments were performed for each calculation. (**A**), the relative expression profile of V-ATPase c subunit in NB and 306 at 0, 24, 48, 72 hpi. (**B**), a and c shows the relative expression profile of V-ATPase c subunit at 24 hpi and 72 hpi, respectively; b and d showes that of B subunit at 24 hpi and 72 hpi, respectively. ^**^ *P*<0.01; ^*^ *P*<0.05.

To determine whether V-ATPase was related to silkworm anti-BmNPV response, we firstly performed comparative real-time PCR using BmNPV-infected larvae midgut cDNA as template (Fig. 1). As shown in Fig. 1A, immediately after virus administration (0 hpi) the expression level of Vc was similar in midguts of NB compared with 306. In NB, it was significantly increased at 24 hpi, the essential period for establishment of BmNPV infection, to more than 3 fold of that at 0 hpi. Then it was sharply decreased at 48 hpi to nearly half of that at 0 hpi (6 folds less than that at 24 hpi), and amazingly it was increased again at 72 hpi to about 2 folds of that at 0 hpi (4 folds of that at 48 hpi). In 306, however, it was firstly increased a little bit at 24 hpi (less than 1 fold), then it was steadily decreased to about half (48 hpi) and 10 folds (72 hpi) less of that at 0 hpi, respectively. This trend was further confirmed by systematic real-time PCR using PBS as control, as shown in Fig. 1B. Similarly, at 24 hpi the expression level of Vc was significantly increased in both NB and BC8 compared to PBS treated silkworms, and was only increased a little bit in 306, and at 72 hpi it was decreased significantly in NB and BC8 but still much higher than corresponding PBS treated silkworm samples. Interestingly, the expression level of VB subunit was also significantly increased at 24 hpi in both NB and BC8, and was also decreased significantly at 72 hpi to normal levels. Together, the real-time PCR showed clearly that the temporal regulation of V-ATPase c and B subunits correlates closely with the BmNPV-invading process, as well as the silkworm self-defending process.

### 2. ATPase assay

**Figure 2:**
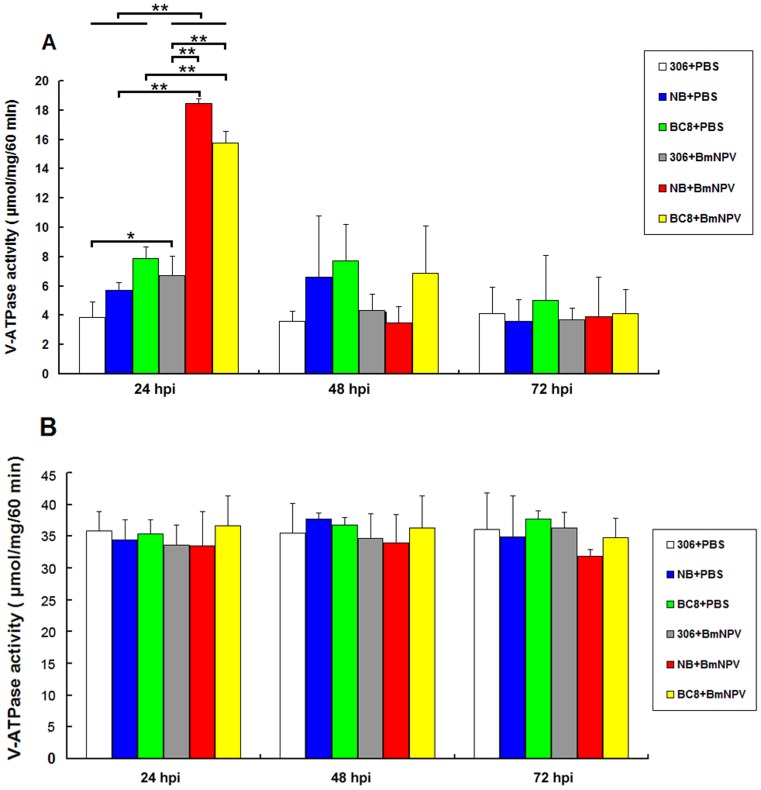
The V-ATPase activity from the BmNPV-resistant silkworm midgut is significantly up-regulated when inoculated with BmNPV, but not for that from midgut plasma membrane. The silkworm strain 306, NB and BC8 were infected with BmNPV, and at different stage of infection the midgut crude extract (**A**) and plasma membrane fractions (**B**) were prepared to measure the V-ATPase activity in triplicates, as described in Materials and Methods. The ATPase is expressed as µmol/mg/60 min. ^**^ *P*<0.01; ^*^ *P*<0.05.

To further investigate whether the up-regulation of expression of V-ATPase c and B subunits correlates with up-regulation of ATPase activity, we determined the V-ATPase activities in midgut crude extracts and midgut plasma membranes isolated from both BmNPV- and PBS-treated larvae. The V-ATPase activity showed a statistically significant increase at 24 hpi in the midgut crude extracts from strain NB and BC8 when challenged with BmNPV, and then was decreased to normal levels, whereas no significant changes could be observed in 306 (Fig. 2, A). Importantly, compared to the PBS treated silkworms, the V-ATPase activity did not show significant difference in midgut plasma membranes purified from both BmNPV-resistant and- susceptible strains (Fig. 2, B). This is consistent with our unpublished data that plasma membrane associated V-ATPase is mainly located in the apical membranes of goblet cells in larvae midgut, not in the columnar cells that are the sites the BmNPV infection takes place [32]. Therefore, the V-ATPase activity in midgut plasma membranes may not necessarily be affected by BmNPV infection. The V-ATPases in endomembranes of midgut columnar cells, represented by those in midgut crude extracts, are the true players involved in silkworm resistance to BmNPV infection. Thus, the ATPase assay showed clearly that the V-ATPases are also spatially regulated. Only those in midgut columnar cells, which directly confront with BmNPV viruses, are significantly up-regulated to control virus infection.

### 3. The effects of transient over-expression of B. mori V-ATPase c and B subunits on BmNPV proliferation

The real-time PCR and ATPase assay suggest that up-regulation of V-ATPase may be required for silkworm anti-BmNPV response. It this is true, the over-expression of V-ATPase may produce similar effects. So we investigated the effects of Vc and VB on virus proliferation by over-expressing them in BmNPV-infected BmN cells.

**Figure 3:**
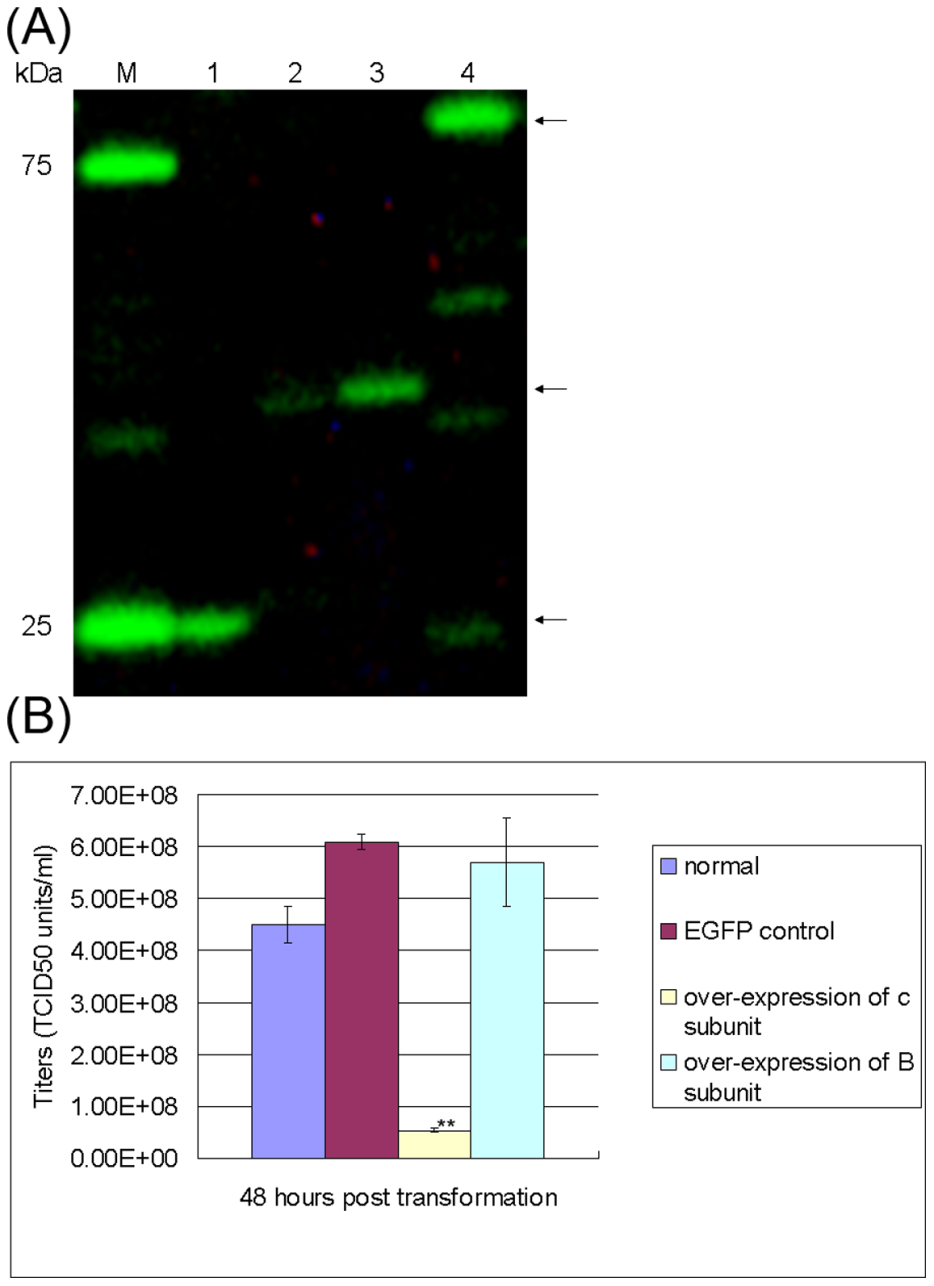
The effects of overexpression of V-ATPase c and B subunits on proliferation of BmNPV. (**A**) SDS-PAGE. The BmN cells were firstly infected with BmNPV virus, then transfected with bacmids to express GFP fused V-ATPase c or B subunit. The total cell lysate was prepared and applied to western blot, and the fluorescence were visualized by FMBIO II (Hitachi, Japan). Lane M, protein molecular weight marker (fluorescent scan, Fermentas); Lane 1, the GFP alone was expressed in BmNPV-infected BmN cells via pFastBacHTb-IE1p-EGFP; Lane 2, the V-ATPase c subunit (EGFP fused) was expressed in BmN cells via pFastBacHTb-IE1p-Vc-EGFP, as well as in BmNPV infected cells (Lane 3); Lane 4, the V-ATPase B subunit (EGFP fused) was expressed in BmNPV infected BmN cells via pFastBacHTb-IE1p-VB-EGFP. The arrows indicate the V-ATPase B subunit, c subunit and EGFP (top-down), respectively. (**B**) The virus titer was calculated as described in Material and Method, and triple experiments were performed for each calculation. Normal indicates the virus titer of BmNPV-infected BmN cells. EGFP control indicates the effect of over-expression of EGFP on the virus titer. ^**^ *P*<0.01; ^*^ *P*<0.05.

**Figure 4:**
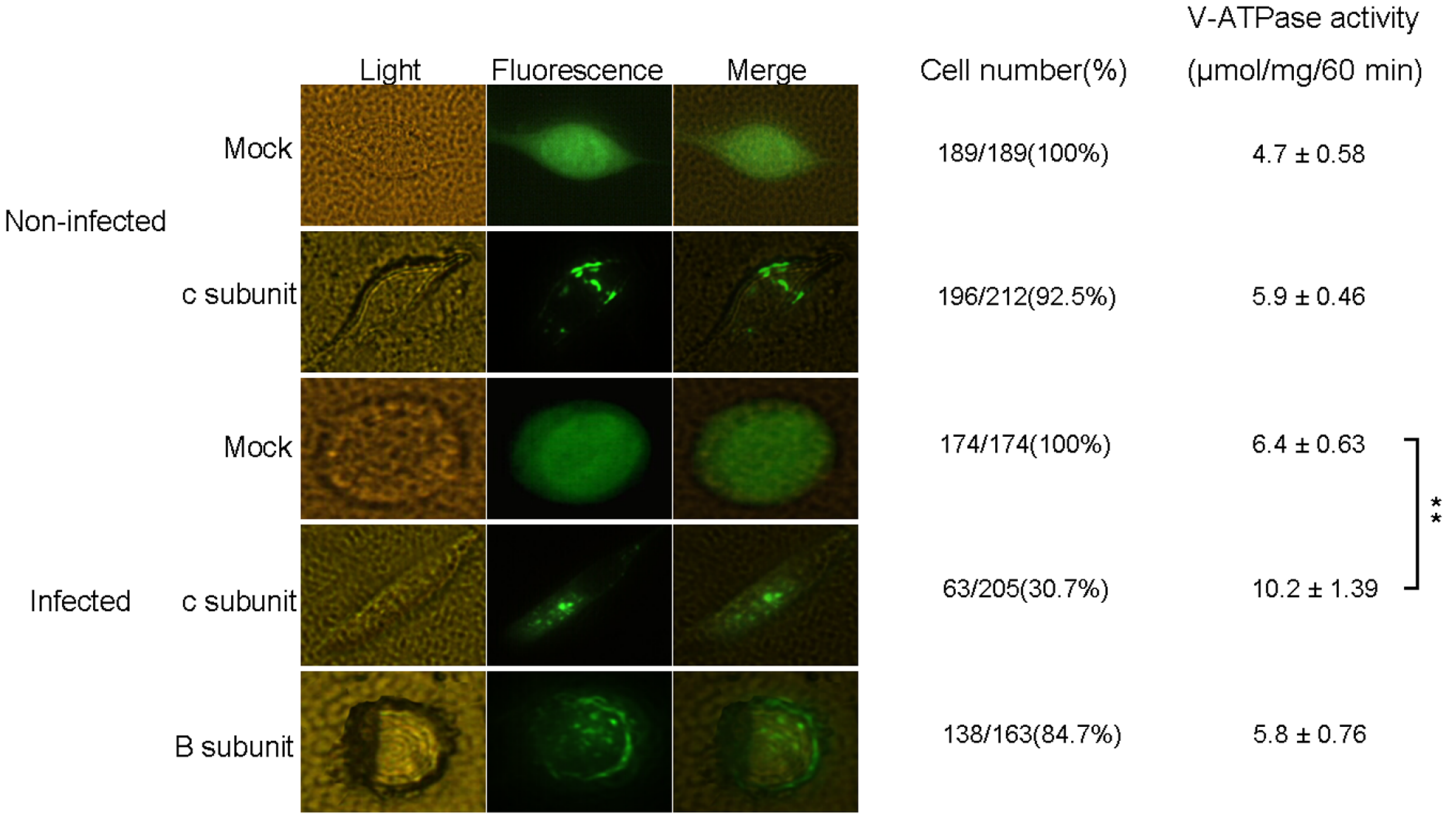
Subcellular localization of over-expressed silkworm V-ATPase c and B subunits in BmNPV-infected BmN cells. The BmN cells were infected with BmNPV, then were transfected with pFastBacHTb-IE1p-EGFP(mock), pFastBacHTb-IE1p-Vc-EGFP and pFastBacHTb-IE1p-VB-EGFP, respectively, and were examined by fluorescence microscopy. The cells with different localization of Vc or VB were counted. ^**^ *P*<0.01.

As shown in western blot (Fig. 3A), the Vc-EGFP fusion protein (about 43 kDa) has been successfully expressed in non-infected BmN cells (lane 2) as well as BmNPV- infected BmN cells (lane 3), and was inclined to localize in some granule-like organelles after BmN cells were infected with BmNPV as revealed by fluorescence microscopy (Fig. 4). The virus titer assay showed that over-expression of Vc conferred a significant inhibitory effect on proliferation of BmNPV (Fig. 3B). Similarly to Vc subunit, the VB-EGFP fusion protein (about 80 kDa) has also been successfully expressed in BmNPV infected BmN cells, as shown by the western blot (lane 5, Fig. 3A), although some low-molecular-weight bands were also observed which could be caused by protein degradation, and was localized mainly in cytoplasm as revealed by fluorescence microscopy (Fig. 4). However, the virus titer assay showed that over-expression of VB did not affect the proliferation of BmNPV (Fig. 3B), although PCR and Western blot analysis (data not shown) clearly showed significant up-regulation of VB subunit induced by BmNPV. It is possible that V-ATPase B subunit is not very stable, which is an interesting discovery and will be discussed in the following section.

## Discussion

It is well known that silkworm endosomal system and V-ATPases play important roles in the infection process of BmNPV [7], [8], [9], [10]. However, up to now it is still elusive whether they may also participate in the silkworm defense response against BmNPV infection. In present study, we firstly provide convincing evidence to support that V-ATPase indeed is also required for silkworm defense response against BmNPV.

We performed real-time PCR and ATPase assay to compare the expression and activity of V-ATPase in BmNPV-susceptible strain 306, -resistant strain NB and a near-isogenic strain BC8. We found that V-ATPase (B and c subunit) was promptly and significantly up-regulated in NB and BC8 midgut, the main site for the first step of BmNPV invasion, but not in that of 306. This up-regulation mainly took place during the first 24 hours after inoculation of virus, the essential period for establishment of BmNPV infection, and then was down-regulated to roughly normal levels, possibly due to elimination of invading BmNPV and the physiological requirement of a balanced ATPase function. In addition, the ATPase activity up-regulated was mainly from the endomembranes of columnar cells in silkworm larvae midgut, not from the goblet cells, which is not the direct site for the first step of BmNPV infection. Thus, the V-ATPase was temporally and spatially regulated in the silkworm resistance response against BmNPV. Because V-ATPase functions to acidify the endosome and lysosome, it is thus tempting to speculate that when encountered with BmNPV, the BmNPV-resistant silkworms may promptly up-regulate the expression and activity of V-ATPase mediated by some unknown mechanism to speed up the acidification and maturation of endosomes and/or lysosomes to quickly render them competent for degradation of invading viruses, which can not be blocked or reversed by BmNPV. The BmNPV-susceptible silkworms still possess some capacity to up-regulate the ATPase (Fig. 1, A), but it is quickly diminished by BmNPV via unknown mechanism, leading to successful infection of BmNPV.

We also transiently over-expressed the V-ATPase B and c subunit in BmNPV infected silkworm BmN cells to examine their effects on BmNPV proliferation. Amazingly, the over-expression of Vc-GFP alone could significantly inhibit the BmNPV proliferation. This could be partially explained by the disruption of cellular entry of BmNPV. Although it is most likely all BmN cells were infected with BmNPV because sufficient amount of virus were administrated as pre-determined to be able to reach an infection rate of 100%, most viruses may still stay in the endosome 1 h after incubation with viruses, which requires normal function of V-ATPase for subsequent virus release into cytoplasm. Thus, over-expression of Vc-EGFP may cause competitive inhibition of V-ATPase activity, leading to blockage of virus cellular entry process and decrease in the virus titer. However, the increased V-ATPase activity after over-expression of Vc-EGFP in BmN cells (Fig. 4) revealed that the over-expressed Vc-EGFP still functions normally. Because V-ATPase c subunit is critical for assembly of functional domain V_0_ [33], and it can activate V_0_ domain for the formation of V_0_ trans-complex and/or control of the fusion pore opening [34], the over-expression of V-ATPase c subunit may prime and/or stabilize the assembly of V-ATPase complex, activate the ATPase activity and speed up the proton translocation process, thus the target endomembrane organelles (endosome, lysosome) can be quickly acidified for efficient degradation of invading viruses. Combined with our PCR analysis and ATPase activity assay, we favor the latter scenario, and subsequent experiments are underway to distinguish these two possibilities.

As for the V-ATPase B subunit, to our surprise, over-expression of it did not show any inhibitory effect on BmNPV proliferation. The B subunit is the component of the V1 domain, and is shown to participate in nucleotide binding and contribute to the ATPase activity of V-ATPases [14], [15]. An interesting discovery in fruit fly showed that VB (vha55) requires stoichometric co-expression of other subunits to be stable [35]. Thus, it is possible that after saturating the V-ATPase complex assembly, the extra amount of over-expressed VB subunit was also not stable and degraded finally, which is also consistent with the observation of multiple bands in western blot (Fig. 3A, lane 4).

To our knowledge, this is the first report that implicates V-ATPase in the silkworm defense response against BmNPV. Consistent with our work, recently the transcription of V-ATPase was also found to be increased in a silkworm strain resistant to silkworm densonucleosis virus (BmDNV-Z), as shown by suppression subtractive hybridization [36]. Therefore, up-regulation of V-ATPase may be a general strategy utilized by silkworms to control virus infection, possibly mediated by prompt acidification of endosome/lysosome to render them competent for degradation of invading viruses. Obviously, more experiments are required to further test this hypothesis. Our work also provides good platform to further investigate the details of the mechanism of silkworm anti-BmNPV response.

## References

[1] Chen KP, Lin CQ, Wu DX (1991) Resistance of the conserved silkworm strains to nuclear polyhedrosis virus disease. Acta Sericol Sin 17: 45–46.

[2] Watanabe H (2002) Genetic resistance of the silkworm, Bombyx mori to viral diseases. Curr Sci 83: 439–446.

[3] Nakazawa H, Tsuneishi E, Ponnuvel KM, Furukawa S, Asaoka A, et al (2004) Antiviral activity of a serine protease from the digestive juice of Bombyx mori larvae against nucleopolyhedrovirus. Virology 321: 154–162. 15033574 10.1016/j.virol.2003.12.011

[4] Lee KS, Kim SR, Park NS, Kim I, Kang PD, et al (2005) Characterization of a silkworm thioredoxin peroxidase that is induced by external temperature stimulus and viral infection. Insect Biochem Mol Biol 35: 73–84. 15607657 10.1016/j.ibmb.2004.09.008

[5] Ponnuvel KM, Nakazawa H, Furukawa S, Asaoka A, Ishibashi J, et al (2003) A lipase isolated from the silkworm Bombyx mori shows antiviral activity against nucleopolyhedrovirus. J Virol 77: 10725–10729. 12970462 10.1128/JVI.77.19.10725-10729.2003 PMC228431

[6] Selot R, Kumar V, Shukla S, Chandrakuntal K, Brahmaraju M, et al (2007) Identification of a soluble NADPH oxidoreductase (BmNOX) with antiviral activities in the gut juice of Bombyx mori. Biosci Biotechnol Biochem 71: 200–205. 17213661 10.1271/bbb.60450

[7] Long G, Pan X, Kormelink R, Vlak JM (2006) Functional entry of baculovirus into insect and mammalian cells is dependent on clathrin-mediated endocytosis. J Virol 80: 8830–8833. 16912330 10.1128/JVI.00880-06 PMC1563848

[8] Blissard GW, Wenz JR (1992) Baculovirus gp64 envelope glycoprotein is sufficient to mediate pH-dependent membrane fusion. J Virol 66: 6829–6835. 1404622 10.1128/jvi.66.11.6829-6835.1992 PMC240187

[9] Leikina E, Onaran HO, Zimmerberg J (1992) Acidic pH induces fusion of cells infected with baculovirus to form syncytia. FEBS Lett 304: 221–224. 1618326 10.1016/0014-5793(92)80623-O PMC7130246

[10] Kingsley DH, Behbahani A, Rashtian A, Blissard GW, Zimmerberg J (1999) A discrete stage of baculovirus GP64-mediated membrane fusion. Mol Biol Cell 10: 4191–4200. 10588652 10.1091/mbc.10.12.4191 PMC25752

[11] Harvey WJ, Nelson N (1992) (eds)V-ATPases. JExpBiol 172: 1–485.

[12] Takase K, Kakinuma S, Yamato I, Konishi K, Igarashi K, et al (1994) Sequencing and characterization of the ntp gene cluster for vacuolar-type Na(+)-translocating ATPase of Enterococcus hirae. J Biol Chem 269: 11037–11044. 8157629

[13] Forgac M (1999) Structure and properties of the clathrin-coated vesicle and yeast vacuolar V-ATPases. J Bioenerg Biomembr 31: 57–65. 10340849 10.1023/a:1005496530380

[14] Nishi T, Forgac M (2002) The vacuolar (H+)-ATPases–nature's most versatile proton pumps. Nat Rev Mol Cell Biol 3: 94–103. 11836511 10.1038/nrm729

[15] Stevens TH, Forgac M (1997) Structure, function and regulation of the vacuolar (H+)-ATPase. Annual Review of Cell and Developmental Biology 13: 779–808. 10.1146/annurev.cellbio.13.1.779 9442887

[16] Beyenbach KW, Wieczorek H (2006) The V-type H+ ATPase: molecular structure and function, physiological roles and regulation. J Exp Biol 209: 577–589. 16449553 10.1242/jeb.02014

[17] Forgac M (2007) Vacuolar ATPases: rotary proton pumps in physiology and pathophysiology. Nat Rev Mol Cell Biol 8: 917–929. 17912264 10.1038/nrm2272

[18] Wagner CA, Finberg KE, Breton S, Marshansky V, Brown D, et al (2004) Renal vacuolar H+-ATPase. Physiol Rev 84: 1263–1314. 15383652 10.1152/physrev.00045.2003

[19] Lü P, Chen KP, Yao Q, Yang HJ (2007) Molecular cloning, bioinformatics analysis and expression profiling of a gene encoding vacuolar H+-ATP synthetase (V-ATPase) c subunit from Bombyx mori. Int J Indust Entomol 15: 115–122.

[20] Yang H, Chen H, Chen K, Yao Q, Zhao G, et al (2009) Characterization and localization of the vacuolar-type ATPase in the midgut cells of silkworm (Bombyx mori). Z Naturforsch C 64: 899–905. 20158164 10.1515/znc-2009-11-1223

[21] Yao Q, Li MW, Wang Y (2003) Screening of molecular markers for NPV resistance in Bombyx mori L. (Lep., Bombycidae). J Appl Ent 127: 134–136.

[22] Maeda S (1989) Gene transfer vectors of a baculovirus, Bombyx mori, and their use for expression of foreign genes in insect cells. CRC Press, Inc, Boca Raton 167–181.

[23] Summers MD, Smith GE (1978) Baculovirus structural polypeptides. Virology 84: 390–402. 622806 10.1016/0042-6822(78)90257-x

[24] Chen HQ, Chen KP, Yao Q, Guo ZJ, Wang LL (2007) Characterization of a late gene, ORF67 from Bombyx mori nucleopolyhedrovirus. FEBS Lett 581: 5836–5842. 18053810 10.1016/j.febslet.2007.11.059

[25] O'Reilly DR, Miller LK, Luckow VA (1992) Baculovirus Expression Vectors: A Laborutory Manual. W H Freeman & Co, New York.

[26] Wong ML, Medrano JF (2005) Real-time PCR for mRNA quantitation. Biotechniques 39: 75–85. 16060372 10.2144/05391RV01

[27] Wieczorek H, Cioffi M, Klein U, Harvey WR, Schweikl H, et al (1990) Isolation of goblet cell apical membrane from tobacco hornworm midgut and purification of its vacuolar-type ATPase. Methods Enzymol 192: 608–616. 2150092 10.1016/0076-6879(90)92098-x

[28] Huss M, Ingenhorst G, Konig S, Gassel M, Drose S, et al (2002) Concanamycin A, the specific inhibitor of V-ATPases, binds to the V(o) subunit c. J Biol Chem 277: 40544–40548. 12186879 10.1074/jbc.M207345200

[29] Weng XH, Huss M, Wieczorek H, Beyenbach KW (2003) The V-type H(+)-ATPase in Malpighian tubules of Aedes aegypti: localization and activity. J Exp Biol 206: 2211–2219. 12771170 10.1242/jeb.00385

[30] Elandalloussi LM, Adams B, Smith PJ (2005) ATPase activity of purified plasma membranes and digestive vacuoles from Plasmodium falciparum. Mol Biochem Parasitol 141: 49–56. 15811526 10.1016/j.molbiopara.2005.02.001

[31] Zhao G, Chen K, Yao Q, Wang W, Wang Y, et al (2008) The nanos gene of Bombyx mori and its expression patterns in developmental embryos and larvae tissues. Gene Expr Patterns 8: 254–260. 18267373 10.1016/j.gep.2007.12.006

[32] Rahman MM, Gopinathan KP (2004) Systemic and in vitro infection process of Bombyx mori nucleopolyhedrovirus. Virus Res 101: 109–118. 15041178 10.1016/j.virusres.2003.12.027

[33] Peters C, Bayer MJ, Buhler S, Andersen JS, Mann M, et al (2001) Trans-complex formation by proteolipid channels in the terminal phase of membrane fusion. Nature 409: 581–588. 11214310 10.1038/35054500

[34] Muller O, Bayer MJ, Peters C, Andersen JS, Mann M, et al (2002) The Vtc proteins in vacuole fusion: coupling NSF activity to V(0) trans-complex formation. Embo J 21: 259–269. 11823419 10.1093/emboj/21.3.259 PMC125839

[35] Du J, Kean L, Allan AK, Southall TD, Davies SA, et al (2006) The SzA mutations of the B subunit of the Drosophila vacuolar H+ ATPase identify conserved residues essential for function in fly and yeast. J Cell Sci 119: 2542–2551. 16735441 10.1242/jcs.02983

[36] Bao YY, Li MW, Zhao YP, Ge JQ, Wang CS, et al (2008) Differentially expressed genes in resistant and susceptible Bombyx mori strains infected with a densonucleosis virus. Insect Biochem Mol Biol 38: 853–861. 18678256 10.1016/j.ibmb.2008.06.004

